# Etoposide-Bound Magnetic Nanoparticles Designed for Remote Targeting of Cancer Cells Disseminated Within Cerebrospinal Fluid Pathways

**DOI:** 10.3389/fneur.2020.596632

**Published:** 2020-11-27

**Authors:** Herbert H. Engelhard, Alexander J. Willis, Syed I. Hussain, Georgia Papavasiliou, David J. Banner, Amanda Kwasnicki, Sajani S. Lakka, Sangyeul Hwang, Tolou Shokuhfar, Sean C. Morris, Bing Liu

**Affiliations:** ^1^Department of Neurosurgery, University of Illinois at Chicago, Chicago, IL, United States; ^2^Department of Bioengineering University of Illinois at Chicago, Chicago, IL, United States; ^3^Department of Medicine, University of Illinois at Chicago, Chicago, IL, United States; ^4^Department of Biomedical Engineering, Illinois Institute of Technology, Chicago, IL, United States; ^5^IMRA America, Inc., Ann Arbor, MI, United States; ^6^Pulse Therapeutics, Inc., St. Louis, MO, United States

**Keywords:** cerebrospinal fluid, glioblastoma, leptomeningeal metastases, lung cancer, magnetic drug targeting, medulloblastoma, nanoparticles

## Abstract

Magnetic nanoparticles (MNPs) have potential for enhancing drug delivery in selected cancer patients, including those which have cells that have disseminated within cerebrospinal fluid (CSF) pathways. Here, we present data related to the creation and *in vitro* use of new two-part MNPs consisting of magnetic gold-iron alloy cores which have streptavidin binding sites, and are coated with biotinylated etoposide. Etoposide was chosen due to its previous use in the CSF and ease of biotinylation. Etoposide magnetic nanoparticles (“Etop-MNPs”) were characterized by several different methods, and moved at a distance by surface-walking of MNP clusters, which occurs in response to a rotating permanent magnet. Human cell lines including D283 (medulloblastoma), U138 (glioblastoma), and H2122 (lung adenocarcinoma) were treated with direct application of Etop-MNPs (and control particles), and after remote particle movement. Cell viability was determined by MTT assay and trypan blue exclusion. Results indicated that the biotinylated etoposide was successfully bound to the base MNPs, with the hybrid particle attaining a maximum velocity of 0.13 ± 0.018 cm/sec. Etop-MNPs killed cancer cells in a dose-dependent fashion, with 50 ± 6.8% cell killing of D283 cells (for example) with 24 h of treatment after remote targeting. U138 and H2122 cells were found to be even more susceptible to the killing effect of Etop-MNPs than D283 cells. These findings indicate that the novel Etop-MNPs have a cytotoxic effect, and can be moved relatively rapidly at physiologic distances, using a rotating magnet. While further testing is needed, intrathecal administration of Etop-MNPs holds promise for magnetically-enhanced eradication of cancer cells distributed within CSF pathways, particularly if given early in the course of the disease.

## Introduction

Seeding of malignant cells into cerebrospinal fluid (CSF) is a cause of severe morbidity and death in patients with metastatic non-CNS tumors (especially lung cancer), as well as those with primary CNS tumors, notably medulloblastoma (1–4). Medulloblastoma cells are notorious for CSF pathway dissemination, which is found in 30–40% of children at initial diagnosis and the majority at recurrence (5–7). Intrathecal chemotherapy, such as with etoposide or methotrexate, has been attempted for patients having disseminated medulloblastoma, and leptomeningeal metastases (LM) from solid tumors (1, 2, 5, 8–11).

Nanotechnology offers the possibility of creating hybrid delivery systems which improve tumor targeting, thereby improving efficacy and decreasing systemic toxicity (12–15). It would be desirable to have a nanoparticle-drug conjugate that could be directed through CSF to target and kill cancer cells, especially early in the course of disease before CSF pathways become obstructed (16, 17). In patients with primary CNS tumors this might extend life; in patients with leptomeningeal metastases (LM) from non-CNS tumors, progression of neurologic symptoms might be delayed.

Rotational magnetic drug targeting (rMDT), which utilizes clusters of magnetic nanoparticles (MNPs) which move across surfaces or down conduits in response to a rotating magnetic field, has recently been shown to be feasible at human-sized distances (16, 18, 19). Here, we present data related to the creation and *in vitro* use of a new two-part MNP consisting of: (1) a AuFe base particle having streptavidin (SA) binding sites, and (2) biotinylated etoposide, which was bound to the base particle. Etoposide was chosen as the therapeutic component due to its ease of biotinylation, and previous intrathecal use. Etoposide is often used in multidrug regimens for medulloblastoma (20–23), and treatment of small and non-small cell lung cancer (24–27). To our knowledge, the creation of such MNPs, and their movement and testing on cancer cells has not previously been described.

## Materials and Methods

### Production of Biotinylated Etoposide- Bound Magnetic Nanoparticles (Etop-MNPs)

**Figure 1:**
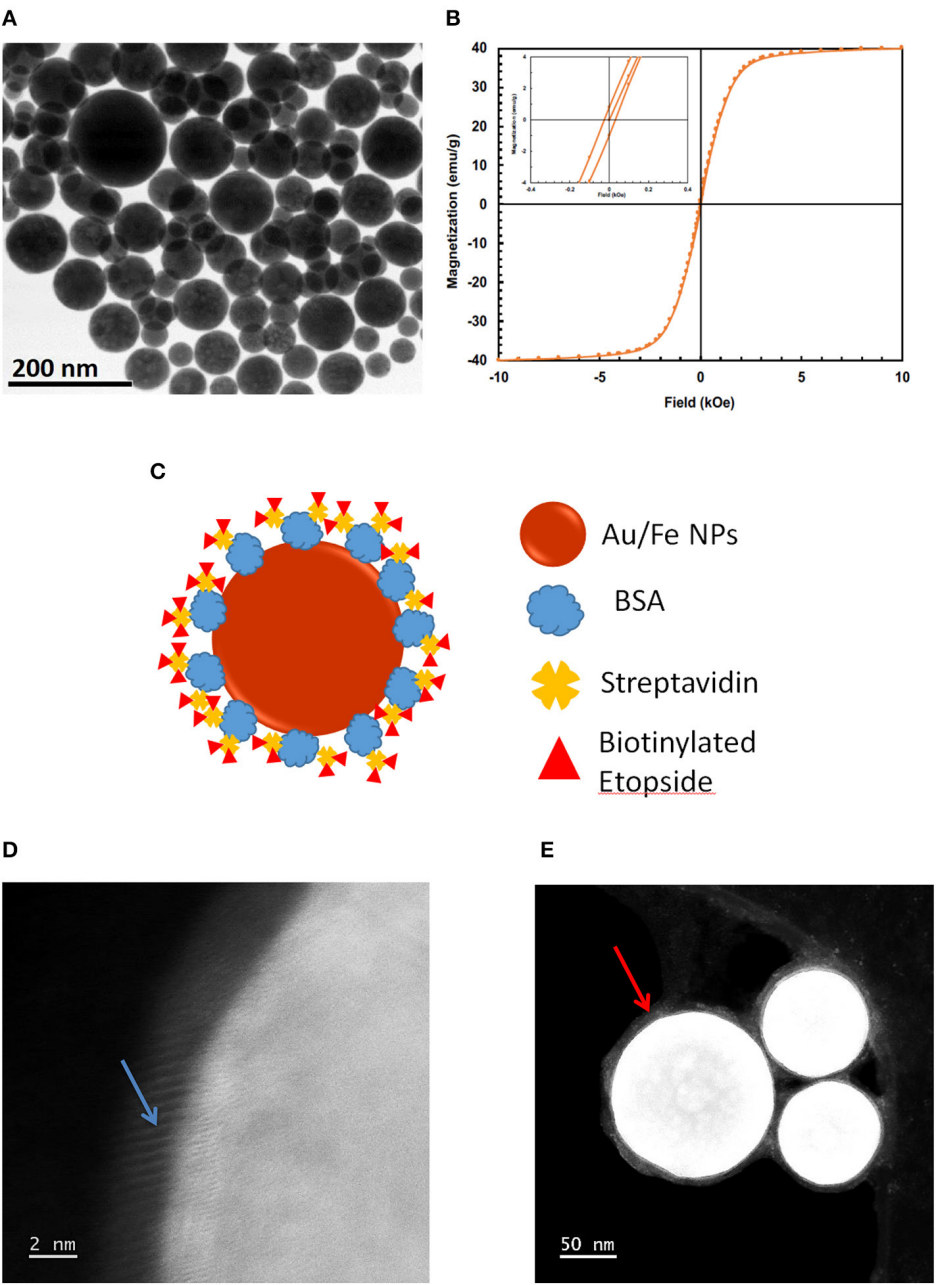
Production of Etop-MNPs. **(A)** TEM of bare MNPs, indicating their size range. **(B)** Magnetization of the bare MNPs, measured with a superconducting quantum interference device (SQUID) magnetometer, showing their superparamagnetic nature. **(C)** Schematic representation of etoposide –bound magnetic nanoparticles “Etop-MNPs.” “Bare MNPs” consist of Au/Fe nanoparticles (the spherical shell) only. “Base MNPs” contain BSA and streptavidin, but do not contain the biotinylated etoposide component (represented by red triangles). **(D,E)** S/TEM images from energy-dispersive x-ray spectroscopy (EDS) studies. **(D)** The surface of a base MNP shown at higher magnification. Here, the alignment of the atomic columns (blue arrow) can be used to determine the elements at the surface of the particle. **(E)** Etop-MNPs showing the biotinylated etoposide coating of the particles (red arrow).

Magnetic gold-iron alloy nanoparticles were manufactured by IMRA America, Inc. (Ann Arbor, MI) using a pulsed-laser ablation method. “Bare MNPs” consist of spherical AuFe alloy nanoparticles only, while “base MNPs” include bovine serum albumin (BSA) and streptavidin linking complex. “Etop-MNPs” add biotinylated etoposide to the base MNPs. Bare MNPs (without streptavidin) vary in diameter and are superparamagnetic, as seen in Figures 1A,B. Figure 1B shows a magnetization curve of bare MNPs measured with a superconducting quantum interface device (SQUID). The inset shows their magnetic coercivity. Base MNPs (with streptavidin) have a biotin binding capacity of 4 × 10^3^ pmol/mg iron.

Etoposide (molecular weight 588.6) was purchased from Cayman Chemical (Ann Arbor, MI) and combined (1:1.1) with iodoacetyl-LC-biotin (Proteochem, Hurricane, UT) and potassium carbonate at RT, according to a method adapted from that of Takami et al. (28) doi: 10.3390/molecules16054278. The phenolic group of etoposide was alkylated with biotin, forming a permanent bond. Biotinylated etoposide was found to have a molecular weight of 971.1. Preparative high pressure liquid chromatography was used for purification, and liquid chromatography—mass spectroscopy and nuclear magnetic resonance confirmed the structure. Etoposide and biotinylated etoposide powders were dissolved in DMSO at 1 mg/ml and stored at −20°C.

For production of Etop-MNPs, 4.5 mg of base MNPs from a stock solution of 25 mg/mL, was combined with 10 μL of 1 mM biotinylated etoposide. Etop-MNP structure is illustrated in Figure 1C. Different “strengths” of Etop-MNPs were made by incubating base MNPs with different molarities of biotinylated etoposide ranging from 20 to 333.3 μM.

MNPs were always vortexed thoroughly, and washed three times with PBS using a LifeSep magnetic separator (Dexter Magnetic Technologies, Elk Grove Village, IL), then suspended in PBS (or other media) with 10 min of sonication prior to use, using the Sonics Vibra-Cell™ (Sonics & Materials, Inc., Newton, CT). To create the standard etoposide AuFe nanoparticles (“Etop-MNPs”), 4.5 mg of base MNPs from a stock solution of 25 mg/mL, was resuspended in 100 μL PBS and combined with 10 μL of 1 mM biotinylated etoposide stock solution (in DMSO) for 30 min at room temperature (RT) with constant shaking. These 1X Etop-MNPs were washed three times in PBS, using the magnetic separator, and suspended in PBS to a final volume of 300 μL, with 100 μL typically being used for an experiment, in triplicate. Etop-MNPs were made fresh for each experiment.

Base MNPs, Etop-MNPs, and a mixture of base MNPs with non-biotinylated etoposide (588.56 g/mol, 5.9 μL) underwent full spectrum (340-850 nm) scans, using a SpectraMax 340PC (Molecular Devices, San Jose, CA). For FITC binding studies [doi: 10.1016/B978-0-12-382239-0.00011-X], 1 mg of base MNPs was incubated in PBS, or 10 μM, 50 μM, 200 μM, or 1 mM biotinylated etoposide for 30 min. Base and Etop -MNPs were suspended in 50 μL of PBS or 1 mg/mL FITC, incubated overnight at 4°C with shaking, then centrifuged, resuspended, sonicated, loaded into a 96-well plate at dilutions of 1:1, 1:2, and 1:5 (displayed), then scanned with excitation at 485 nm and emission at 525 nm.

### Energy-Dispersive X-Ray Spectroscopy (EDS) and Electron Microscopy

Base and Etop -MNPs were prepared for scanning transmission electron microscopy (S/TEM) and energy dispersive x-ray spectroscopy (EDS) analysis with 10 washes of OmniTrace® Ultra picopure water (EMD Millipore Corperation, Billerica, MA) to remove contaminants. MNPs were drop cast onto Lacey Carbon Grids (Electron Microscopy Sciences, Hatfield, PA) and dried sterilely for 24 h. MNPs were visualized using a JEOL-ARM200CF electron microscope (JEOL USA, Inc., Glen Ellyn, IL) operated at 200 keV. EDS line scan data was collected 0–10 keV with 10 eV per channel and dwell time of 15 μs.

Samples for scanning electron microscopy (SEM) were dehydrated with ethanol followed by hexamethyldisilazane. Glass coverslips were adhered to aluminum mounts, then sputter-coated with 6.0 nm of Pt/Pd in a low pressure argon atmosphere. Surface morphology was examined using a Hitachi S-3000N Variable Pressure SEM (Hitachi America, Ltd., Santa Clara, CA) using secondary and backscatter detectors. MNPs have a high electron density and therefore appear bright on the images. For transmission electron microscopy (TEM), cells were fixed in 2.5% glutaraldehyde, dehydrated by an ethanol series, and embedded in epoxy resin. Ultra-thin sections (70 nm) were collected and stained with uranyl acetate and lead citrate. Specimens were examined using a JEOL JEM-1220 transmission electron microscope at 80 kV.

### MNP Characterization

Hydrodynamic radius determinations for bare, base, and Etop -MNPs were performed using a nanosizer instrument (Nanosight LM10, Malvern, UK) equipped with nanoparticle tracking analysis software (NTA v3.0, Malvern). MNPs were diluted to 10 ug/ml, sonicated, and loaded bubble-free. Motion was tracked for 30 s at RT (pH 7.0–7.4), after adjusting the detection threshold and eliminating noise.

Dynamic light scattering (DLS) was used to determine the physical radius of MNPs on a NanoDLS instrument (Brookhaven Instruments Corp., Holtsville, NY). Dilutions of MNPs were prepared with sonication and vortexing, then analyzed for 45 sec. Average particle diameter was determined from the log-normal distribution of intensity.

The diameter, zeta potential, and polydispersity index (PDI) of the various MNPs were also determined using the Zetasizer Nano ZSP (Malvern). For these experiments, MNPs were prepared at 1 mg/ml in PBS, dH_2_O, or 10 mM sodium phosphate buffer (NaP), for 24 and 48 h. For zeta potential, three measurements over 30 s were taken. Eight measurements over 30 s were taken to determine size and PDI.

### Velocity of MNPs in Response to the Rotating Magnet

Base and Etop -MNPs were tested for movement at a distance by means of the “surface walking” phenomenon occurring in response to a rotating neodymium-boron-iron permanent magnet, as described previously (16, 18, 19). Acrylic trays for measuring the velocity of MNPs in response to the rotating magnet have also been previously described (16, 18, 19). Trays were cleaned and between each use with 100% ethanol and UV light, then rinsed thoroughly with dH_2_O. 2 mL of media was loaded into each tray lane.

Digital videos of MNPs being moved by the magnet through PBS or artificial CSF (Harvard Apparatus, Hollistan, MA) were recorded using an Olympus SZ-12 camera (Center Valley, PA). The tray was positioned so MNPs were running directly at the rotating magnet (“pull position”), starting at a distance of 15 cm. In this position, MNPs are being moved both by magnetic attraction and the rotational force which causes surface walking. 100 μL of MNPs was added to the starting points in the lanes. Digital recording analysis was performed using MATLAB's Video Viewer application (MathWorks, Natick, MA).

### Culture, Treatment, and Viability Testing

The D283 (human medulloblastoma), U138 (human glioblastoma), and H2122 (human lung adenocarcinoma) cell lines were recently obtained from the American Type Culture Collection (Manassas, VA) and maintained in low passage using standard tissue culture technique with 1% Penicillin/Streptomycin solution (ThermoFisher, Waltham, MA), 1% Ciprofloxacin solution (Corning Inc., Corning, NY), and 10% fetal bovine serum (ThermoFisher). D283, U138, and H2122 cells were grown in Minimal Essential Medium with Earle's Salt, Dulbecco's Modified Eagle Medium, and RPMI 1640, respectively (ThermoFisher). Cells were seeded into standard multiwall plates with coverslips when performing SEM, and without coverslips when performing MTT assay or trypan blue testing. Morphologic responses and viability of cells in response to etoposide, biotinylated etoposide, base MNPs, and Etop-MNPs, were studied by phase contrast light microscopy using an inverted microscope (Nikon Corp., Tokyo, Japan), an EVOS FL Auto 2 microscope (Fisher Scientific, Waltham, MA), and SEM (as described above). The MTT assay was performed in 12-well plates per Abcam (Cambridge, UK) protocol, as previously described (16).

For cytotoxicity analysis by trypan blue exclusion, the supernatant from the well (including any dead cells) was transferred into a 15 mL Falcon tube (Corning, Inc., Corning, NY) and 1 mL of media was added per well in to harvest the remaining cells. Cells were detached with gentle use of a cell scraper and added to the 15 mL tube, which was centrifuged at 1,800 rpm for 5 min. Cell pellets were resuspended in 1 mL of media. 100 μL of cell suspension was added to 400 μL of trypan blue. 10 μL of trypan blue cell suspension was then transferred to a hemocytometer, and live and dead cells were counted, with counts being performed 5 times per group.

### Cytotoxic Effect of MNPs After Remote Targeting (Dynamic Studies)

Translational (i.e., dynamic) experiments were performed to determine whether or not Etop-MNPs could be transported down the length of the acrylic tray in response to the rotating magnet and maintain a chemotherapeutic effect. Sixty μL of base MNPs alone vs. 60 μL of 1X, 2X, and 5X Etop-MNPs were loaded 15 cm from the magnet center. The rotating magnet was turned on (“activated”) for 5 min to ensure all MNPs had traveled the full distance. MNPs were removed from the end of each lane and applied to wells of 12-well tray containing monolayers of D283 or H2122 cells which were seeded at 10^5^ cells/well and grown for 24 hrs. After treatment for 2 h (H2122) or 24 h (D283), cytotoxicity was analyzed by trypan blue exclusion.

### Statistical Analysis

Data are expressed as mean ± SEM with *n* > 3. Statistical significance for the DLS, UV-vis absorbance, and velocity determinations was determined using two-factor ANOVA with replication. A value of *p* < 0.05 was considered to be statistically significant.

## Results

### MNP Characterization: S/TEM, EDS, UV-vis, and Size

**Figure 2:**
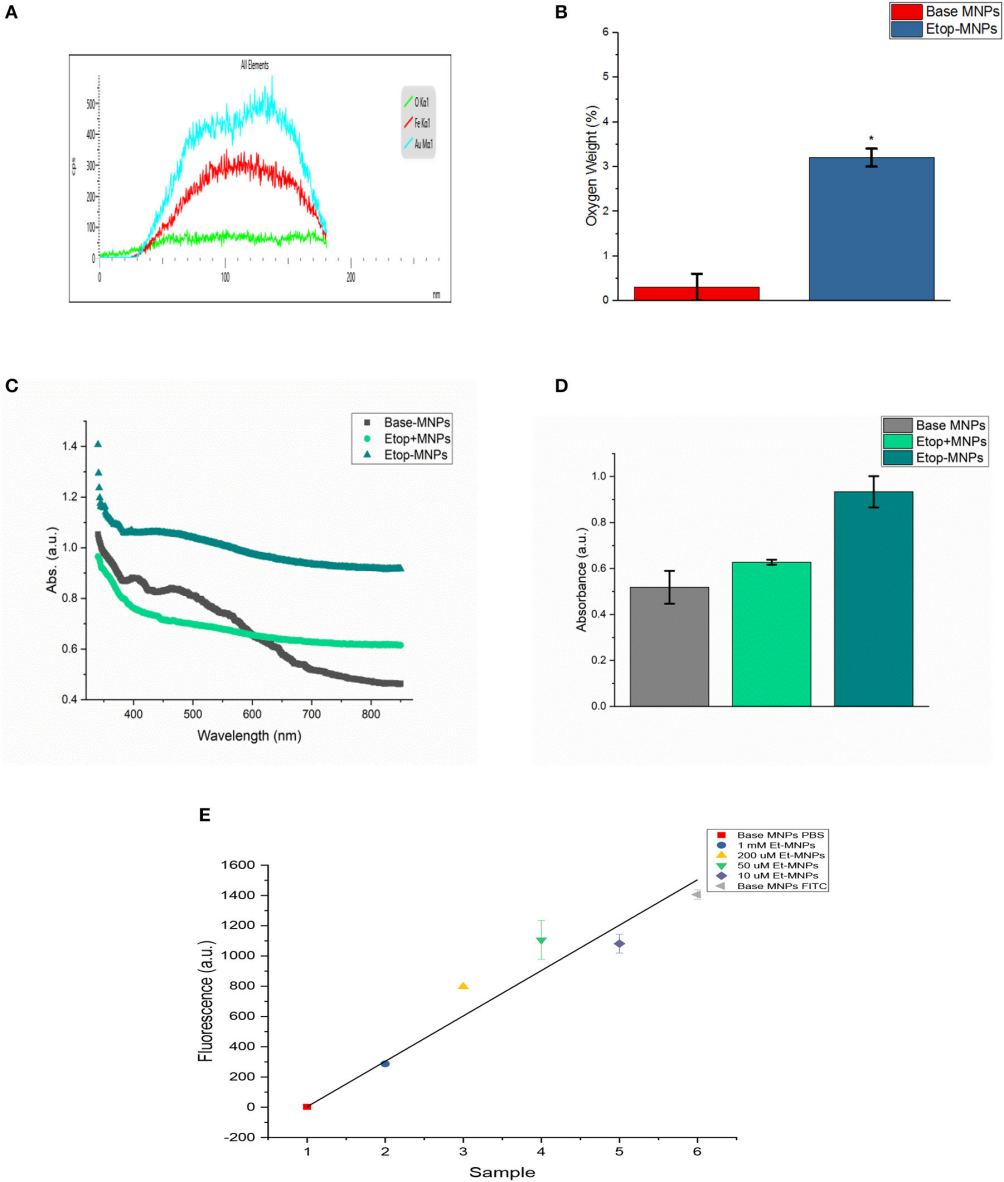
Characterization of Etop-MNPs. **(A)** Representative EDS data histogram, showing the percentage of weight by element for a single, whole Etop-MNP. **(B)** Bar graph showing the higher oxygen content of the Etop-MNPs, confirming that etoposide has bound to the base MNPs. **(C)** UV-visible spectra for base MNPs, a mixture of etoposide with base MNPs and the bound Etop-MNPs, from 340 to 850 nm. **(D)** Bar graph showing the absorption at 700 nm, which highlights the differences between the particles. **(E)** FITC loading of various strengths of Etop-MNPs, showing the saturation of binding sites by biotinylated etoposide. For **(B,D,E)**, data are expressed as mean ± SEM with *n* = 3.

Etop-MNPs were studied by multiple techniques to compare them to their “bare” and “base” MNP counterparts. S/TEM images from the EDS studies are shown in Figures 1D,E. The surface of a base MNP is shown at high magnification in Figure 1D. The alignment of the atomic columns can be used to determine the elements at the particle surface (arrow). Etop-MNPs are shown in Figure 1E. Here, the coating of the particles is clearly seen. EDS confirmed that Etop-MNPs have a higher oxygen content than base MNPs, as seen in Figure 2A (data histogram) and Figure 2B (summary bar graph). This confirms etoposide is bound to the base MNPs. Image J analysis of the S/TEM particles showed that the mean diameters of bare, base and Etop –MNPs were 73.1 ± 5.5, 94.3 ± 16.5, and 124.7 ± 12.6 nm, respectively.

Etoposide was also shown to be successfully bound to the base particle by UV-visible spectroscopy. Base MNPs, etoposide drug mixed with base MNPs, and Etop-MNPs underwent a full spectrum (340–850 nm) scan. Data is shown in Figure 2C. The absorbance of Etop-MNPs was seen to be greater at all wavelengths, indicating the difference from base particles alone, and base particles mixed with etoposide without the biotin-streptavidin linkage. For generating the bar graph (shown in Figure 2D), 700 nm was used. Quantitative binding studies were then performed in order to examine the change in FITC fluorescence as the molarity of biotinylated etoposide mixed with base MNPs was increased from 10 μM to 1 mM. As would be expected with satisfactory binding of biotinylated etoposide to the base MNPs, particles incubated with the minimum strength of biotinylated etoposide (10 μM), had the maximum FITC fluorescence. Conversely, MNPs incubated with the maximum strength of biotinylated etoposide (1 mM) had minimal fluorescence of any bound FITC, as shown in Figure 2E.

**Figure 3:**
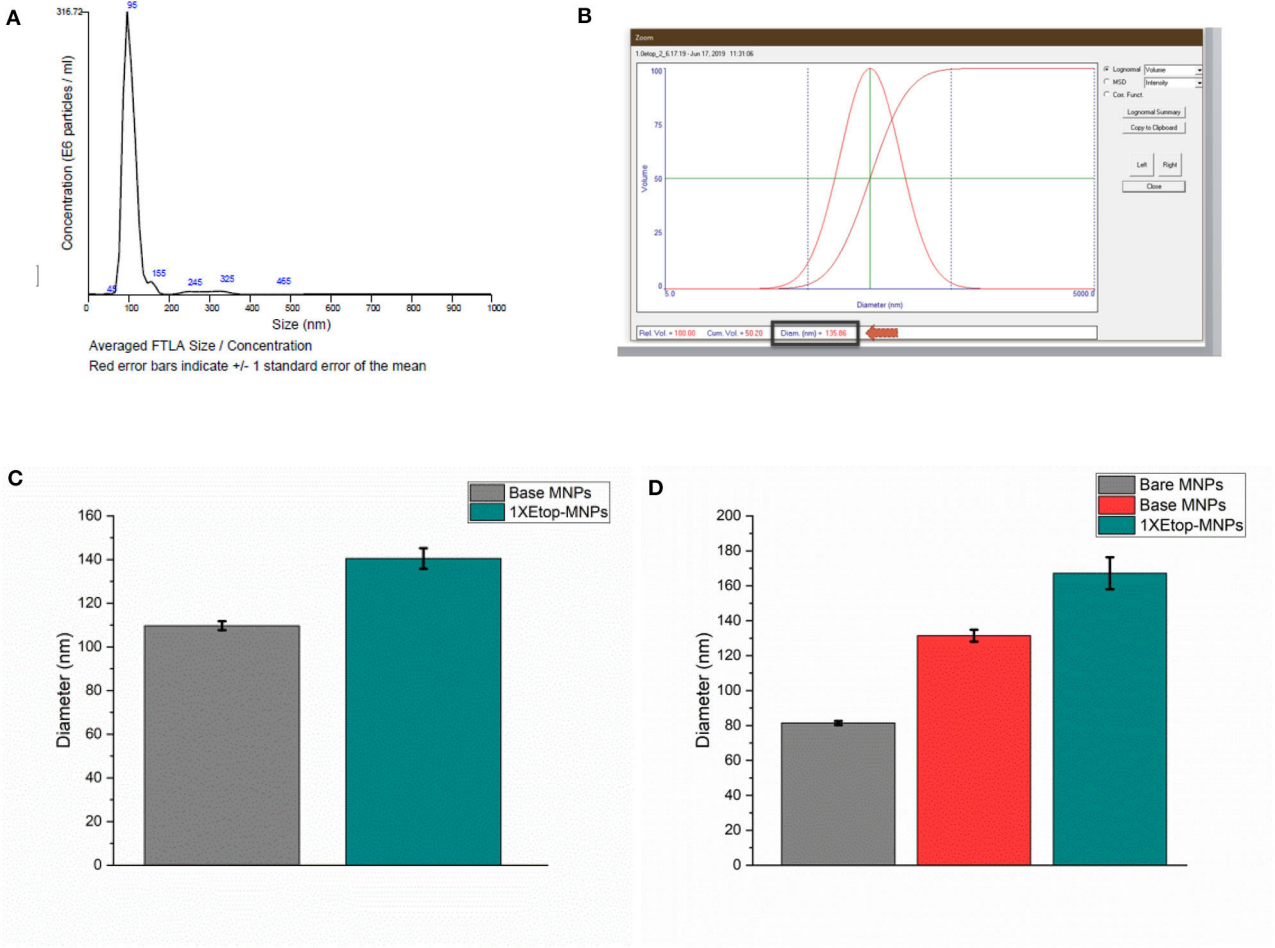
Analysis of base versus Etop -MNP diameters. **(A)** Representative histogram from the Nanosight instrument, which indicates particle size by Brownian motion. **(B)** Representative data (“screen shot”) from the NanoDLS instrument, which determines particle size by dynamic light scattering. **(C)** Bar graph showing mean diameter of base MNPs versus Etop-MNPs suspended in PBS, as determined by the NanoDLS instrument. The Etop-MNPs are slightly larger due to the coating with biotinylated etoposide. **(D)** Bar graph showing independent analysis of base MNP versus Etop-MNP particle size using the Zetasizer instrument, in sodium phosphate solution, in which the particles showed a higher stability. For **(C,D)**, data are expressed as mean ± SEM with *n* = 3.

Evaluation of MNP size and etoposide binding (in addition to S/TEM) was performed using multiple independent techniques, including nanoparticle tracking analysis, dynamic light scattering (DLS), and DLS combined with electrophoretic light scattering. MNP sizes were quantified using nanoparticle tracking analysis in order to individually and simultaneously measure the Brownian motion of each particle. Particle diffusion was then converted to particle hydrodynamic radius using the Stokes-Einstein equation. A representative histogram is shown in Figure 3A. Base MNPs were found to have a mean diameter of 102.6 nm using this method, whereas Etop-MNPs had a mean diameter of 128.1 nm. A representative NanoDLS histogram is shown in Figure 3B.

Using DLS, base MNPs were found to have a mean diameter of 104.0 ± 3.5 nm, whereas Etop-MNPs had a mean diameter of 151.6 ± 6.4 nm. This difference is shown in the bar graph given in Figure 3C and was found to be statistically significant (*p* < 0.5). MNP suspension in different types of solvents did affect such determinations. MNP measurements using the Zetasizer instrument, comparing bare, base and Etop -MNPs in 10 mM sodium phosphate are shown in Figure 3D. Here the effective MNP diameters were larger, but in accordance with data from the other methods. Specifically, the mean diameter of the Etop-MNPs was 27.2% larger than that of base MNPs, indicating the binding of the biotinylated etoposide.

### MNP Characterization: Zeta Potential, and Polydispersity Index (PDI)

**Figure 4:**
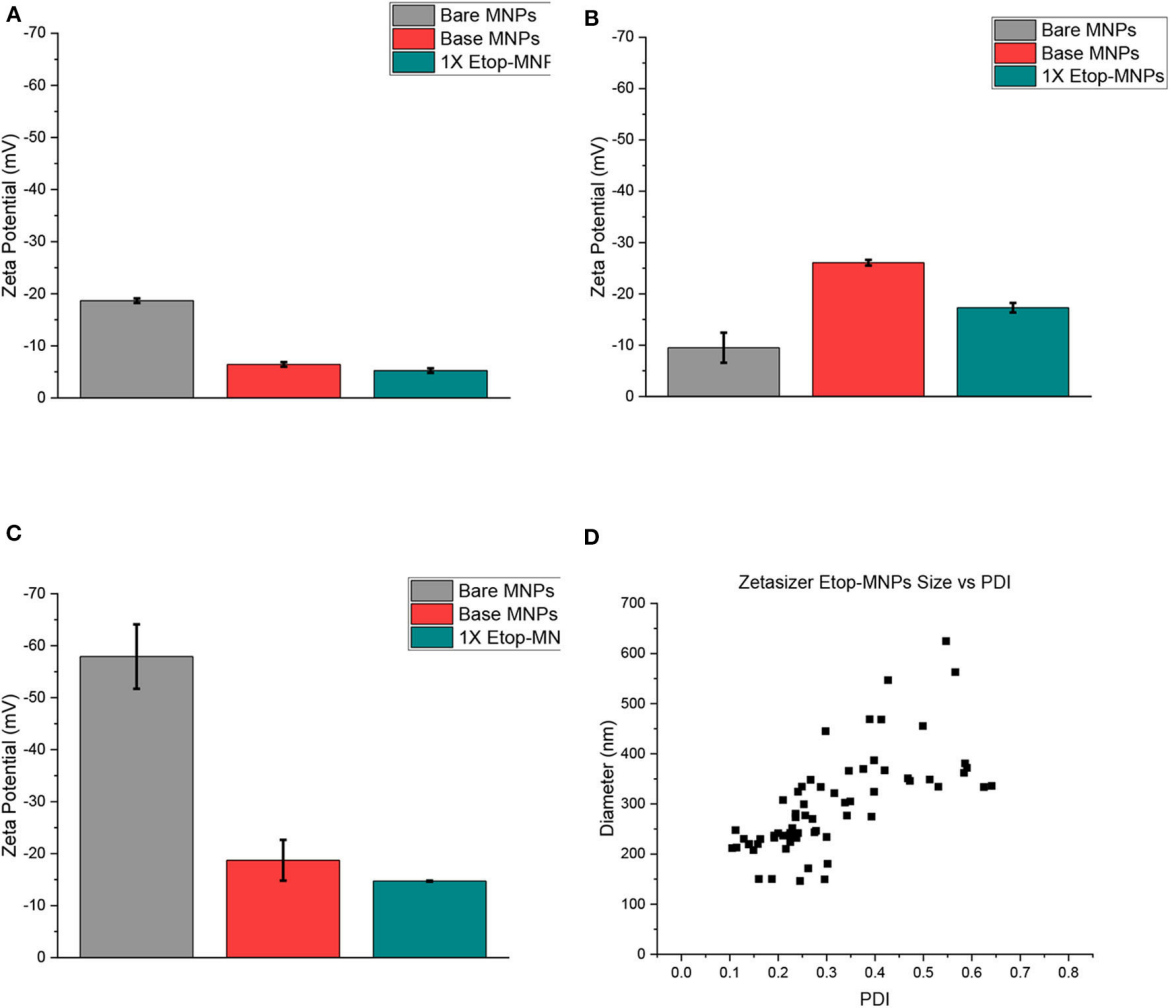
Analysis of MNP zeta potential and polydispersity. **(A–C)** Bar graphs of zeta potential data, obtained from the Malvern Zetasizer instrument for bare, base, and Etop-MNPs at 24 h in PBS **(A)**, dH_2_O **(B)**, and 10 mM NaP **(C)**. **(D)** Scatter plots of polydispersity data, for Etop-MNPs. The polydispersity index (PDI) is shown along the x-axis, and aggregate diameter is shown along the y-axis. For **(A–C)**, data are expressed as mean ± SEM with *n* = 3.

Zeta potential was used to evaluate surface charge and stability characteristics of base MNPs and Etop-MNPs, in different solvents (PBS, dH_2_O, and NaP), for different time periods (1, 24, and 48 h). These data are shown in Figures 4A–C for the 24-h time period, with greater negative values indicative of increasing particle stability. Etoposide, biotin, and streptavidin all have a neutral charge. Bare MNPs had the highest zeta potential. At 24 h Etop-MNPs were found to be highly stable with stability dependent on solvent type. Etop-MNP stability was highest in NaP, followed by dH_2_O, and PBS (the most ionic solution), as shown in Figures 4A–C. The differences in zeta potential values for base MNPs vs. Etop-MNPs, in PBS and NaP, were found to be statistically significant (*p* < 0.05 by ANOVA). These data are consistent with a uniform distribution of binding of the available streptavidin sites by the biotinylated etoposide.

The polydispersity index (PDI) of the different MNPs was evaluated using the Zetasizer, as shown in Figure 4D for Etop-MNPs. A PDI value <0.1 indicates a narrow size distribution and monodisperse particles. A value of 0.1–0.2 indicates a broader size distribution and PDI >0.2 indicates particle aggregation with the formation of clusters. While both base and Etop -MNPs are subject to agglomeration, the data indicated a difference in PDI for base MNPs vs. Etop-MNPs, again confirming their individual identities. The larger clusters may be forming either through van der Walls interaction, hydrophobic interactions, and/or flocculation [doi: 10.1016/B978-0-08-100557-6.00003-1]

### Velocity of Etop-MNPs in Response to the Rotating Magnet

**Figure 5:**
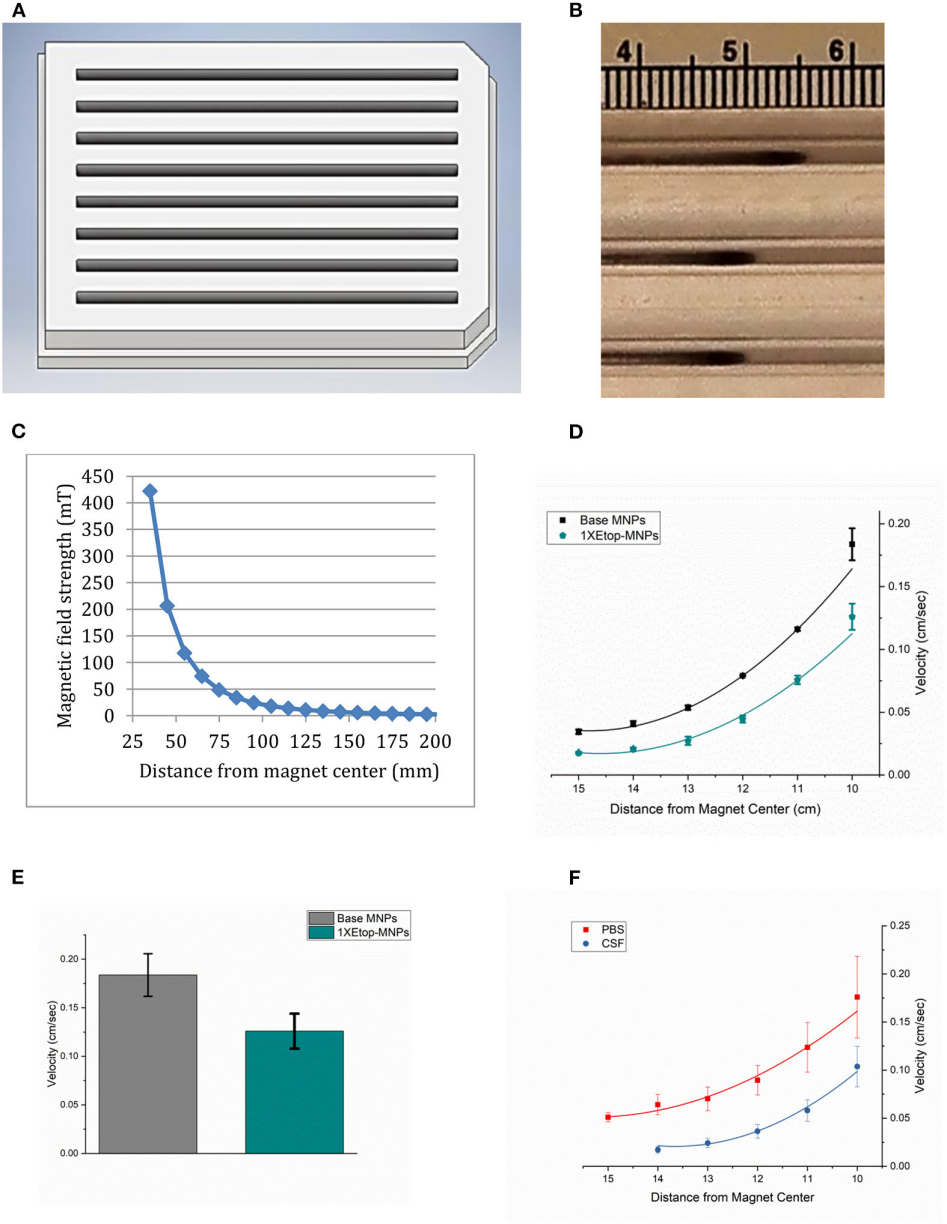
MNP velocity determinations. **(A)** Three-dimensional rendering of the acrylic tray used to measure MNP velocity. MNPs are loaded at one end of the tray and pulled to the other end over a 10 cm distance, by the action of the rotating magnet. This enables testing their relative velocities, and their ability to transport etoposide over a distance, not just by direct application onto cells. Videos are made, which allows the velocities to be calculated. **(B)** Photograph of Etop-MNPs moving down the lanes of the MIRT tray in PBS, in response to the rotating magnet. **(C)** Plot of magnetic field strength as a function of distance from the magnet. **(D)** Velocity of base MNPs vs. Etop-MNPs (cm/sec) in centimeter increments. Adding etoposide slowed down the MNPs by ~31.5%. **(E)** Bar graph showing the maximum velocities of base MNPs and Etop-MNPs. **(F)** Velocity of MNPs in PBS versus artificial CSF. The MNPs moved more slowly in CSF. For **(D–F)**, data are expressed as mean ± SEM with *n* = 3.

While isolated bare, base and Etop -MNPs are superparamagnetic, aggregates of MNPs form in response to a magnetic field. These aggregates rotate and surface walk in response to a rotating permanent magnet (or oscillating electromagnetic field). Figure 5A shows a rendering of the acrylic tray used to measure MNP velocity. MNPs are loaded at one end of the tray and pulled to the other end, by the action of the rotating magnet. MNP velocities are determined by videography, and the tray can be used to evaluate the MNPs ability to transport a drug (here, etoposide) over a distance, not just by direct application onto cells. Figure 5B shows a photograph of Etop-MNPs moving down lanes of the tray in PBS, in response to the rotating magnet. Bare MNPs were found to move faster than base MNPs, and a 50:50 mixture of bare and base MNPs. Figure 5C shows a plot of the magnetic field strength as a function of the distance from the center of the rotating magnet.

The velocity of base MNPs vs. Etop-MNPs (in cm/sec) in centimeter increments, is shown in Figure 5D. Etop-MNPs were found to be slower, by ~31.7%. A bar graph showing the maximum velocities of base MNPs and Etop-MNPs, which occurred closest to the magnet, is given in Figure 5E. Etop-MNPs reached a maximum velocity of 0.13 ± 0.018 cm/s. Both types of particles accelerate as they near the magnet, due to the combination of rotational force (with surface traction), and magnetic attraction. The velocity of MNPs in PBS vs. artificial CSF was also studied, and this data is shown in Figure 5F. MNPs moved more slowly in artificial CSF than in PBS. Etop-MNP clusters were also somewhat more dispersed in artificial CSF, but still traveled at a clinically-relevant speed of ~3 cm/min, depending on the distance from the magnet.

### Effect of Etoposide and Biotinylated Etoposide on D283, U138, and H2122 Cells

**Figure 6:**
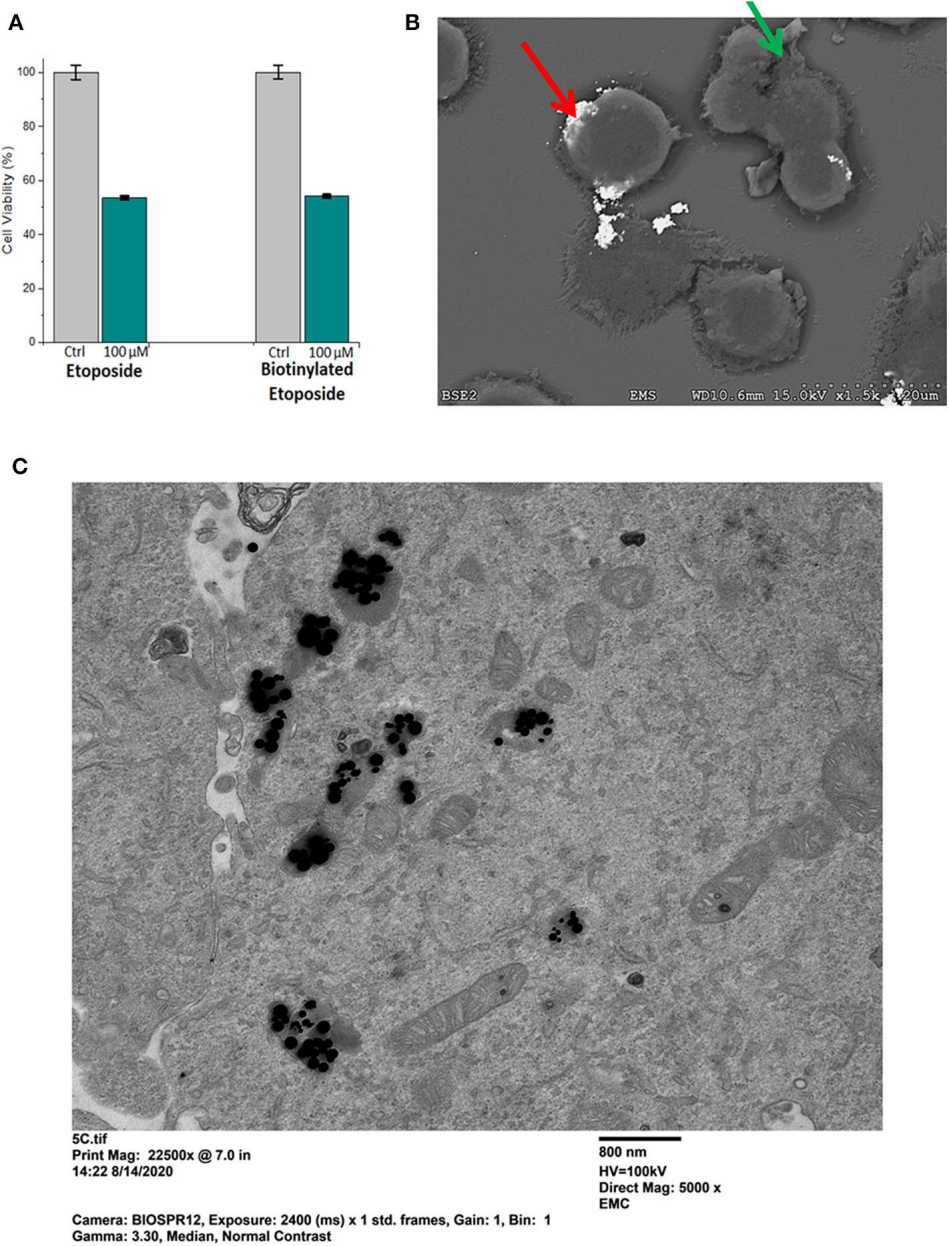
Potency of biotinylated etoposide, and binding and uptake of Etop-MNPs. **(A)** Comparison of the potency of biotinylated etoposide, to etoposide alone, using the MTT assay. D283 cells were incubated for 24 h in 100 μM etoposide vs. 100 μM biotinylated etoposide. Biotinylation did not compromise the therapeutic effect of the drug, and made it amenable to binding to the base MNPs through the streptavidin sites. **(B)** Scanning electron microscope (SEM) pictures of H2122 cells, showing the binding of Etop-MNPs. MNPs appear very bright using this technique (red arrow). Cells show blebbing, indicating the cytotoxic effect of the etoposide (green arrow). **(C)** Transmission electron microscope (TEM) pictures of D283 cells, showing the uptake of Etop-MNPs. MNPs appear to be black using this technique, and can be seen singly and in clusters.

Etoposide was biotinylated in order to specifically bind it to the streptavidin -conjugated superparamagnetic gold-iron alloy base nanoparticles. Potency of biotinylated etoposide alone, in comparison to native etoposide, was studied first (i.e., prior to binding to nanoparticles) using the MTT assay. Results are shown in Figure 6A, for D283 cells incubated for 24 h in 100 μM etoposide or 100 μM biotinylated etoposide, in comparison to control cells not treated with chemotherapy. Lower doses of etoposide and biotinylated etoposide (1–50 μM) were tested in preliminary studies, but (as expected) did not have as potent an effect. While biotinylated etoposide is a larger compound, it was found to have a cytotoxic effect similar to that of native etoposide when cells were treated with equivalent μM concentrations. Testing of H2122 and U138 cells also showed that biotinylated etoposide was comparable to etoposide, but with an even greater cytotoxicity over a shorter time period (79.7 ± 1.0% for U138 cells after a 2 h treatment time). Biotinylation of etoposide therefore did not disrupt its chemotherapeutic effect, making it a suitable drug for binding to base MNPs.

### Cellular Binding, Uptake, and Cytotoxicity of Etop-MNPs - EM and MTT Studies

The effect of Etop-MNPs on cultured cells in 12-well plates was studied, using SEM and MTT assay. Cells were directly treated with base MNPs and Etop-MNPs, in comparison to untreated control cells. An SEM image of Etop-MNPs binding to H2122 cells are shown in Figure 6B. The Etop-MNPs appear very bright using this technique. Cells showed blebbing (indicated by a green arrow), showing the cytotoxic effect of the Etop-MNPs. Analysis by TEM indicated the internalization of Etop-MNPs by cancer cells. A TEM image of D283 cells treated with Etop-MNPs is shown in Figure 6C. Multiple Etop-MNPs (singly and in clusters) are seen within the cells.

**Figure 7:**
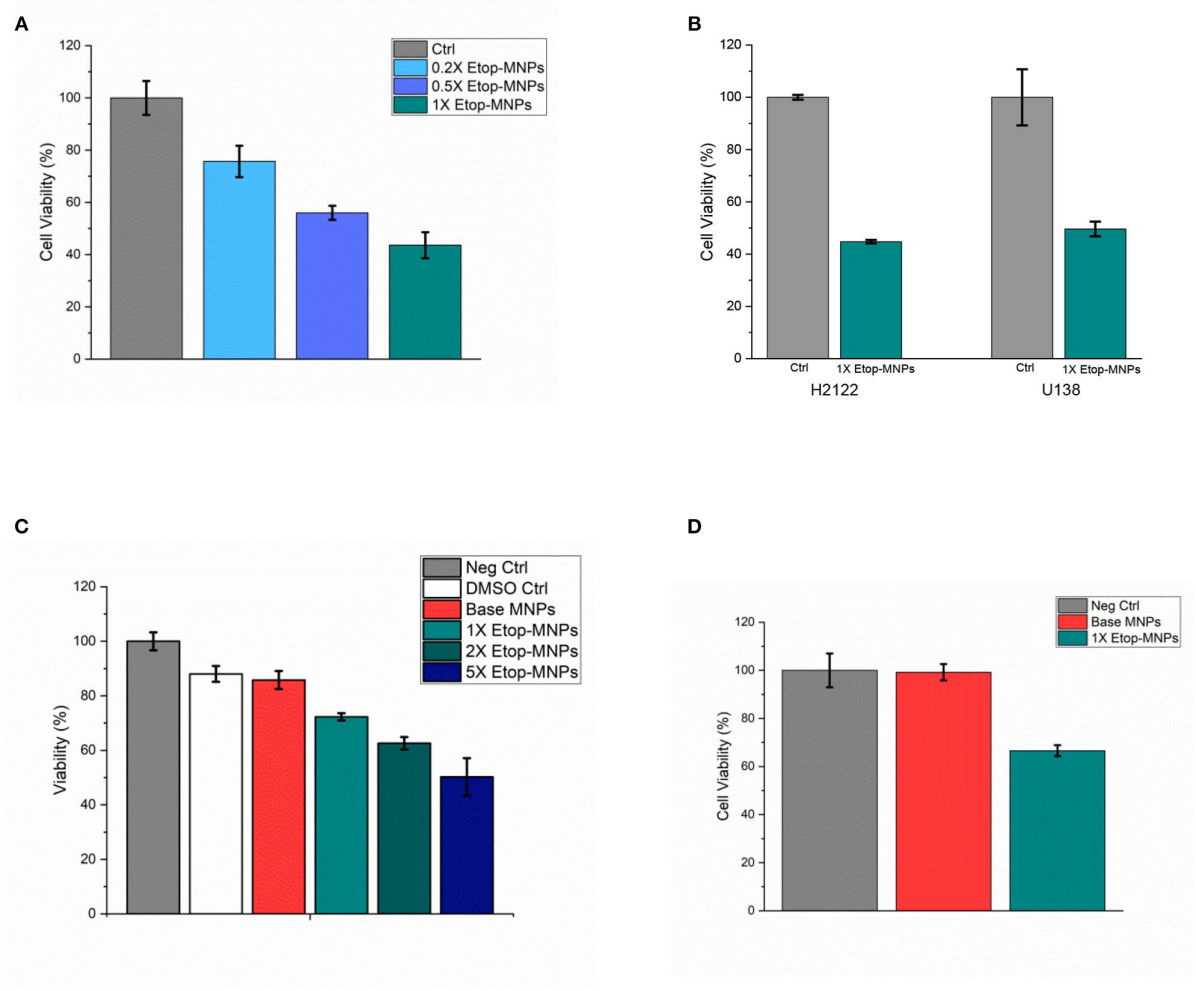
Cytotoxic effect of Etop-MNPs. **(A)** Cytotoxic effect of Etop-MNPs of different strengths applied directly to D283 cells for 24 h in 12 well plates, as determined by MTT assay. **(B)** Cytotoxic effect of 1X Etop-MNPs on H2122 and U138 cells, applied directly for 2 h in 12 well plates, as determined by MTT assay. **(C)** Effect of Etop-MNPs of different strengths, applied to D283 cells for 24 h after translation down the MIRT tray. Etop-MNPs demonstrated a killing effect on cancer cells which was dose-dependent. **(D)** Effect of 1X Etop-MNPs (vs. untreated control cells and base MNP treatment), applied to H2122 cells for 2 h after translation down the MIRT tray. Etop-MNPs were seen to retain their killing effect on the cancer cells after translational movement. For **(A–D)**, data are expressed as mean ± SEM with *n* = 3.

When studied by MTT assay, Etop-MNPs decreased the viability of D283 cells to 43.6 ± 5.0% that of controls, with 24 h of treatment. A dose-dependent cytotoxic effect of increasing strengths (0.2X, 0.5X, and 1X) of Etop-MNPs was found, as shown in Figure 7A. These results were confirmed using trypan blue exclusion, as an independent technique. As was seen with etoposide alone, H2122 and U138 cells were even more sensitive to the killing effect of Etop-MNPs. With 2 hours of treatment, Etop-MNPs decreased cell viability to 44.8 ± 0.7% in H2122 cells, and 49.6 ± 2.8% in U138 cells. These results are illustrated in Figure 7B.

### Cytotoxic Effect of Etop-MNPs After Remote Delivery

Since Etop-MNPs were designed with the hope that they could ultimately be used in patients for targeted chemotherapy delivery, different strengths of Etop-MNPs (and base MNPs) were moved at human-sized distances by the rotating magnet in the acrylic trays. As in experiments above, “strength” of the Etop-MNPs was defined according to the molarity of biotinylated etoposide that was originally incubated with the base MNPs. The trans-located MNPs were transferred from the acrylic tray lanes to the media of cancer cells seeded into multi-well plates. This was to determine if the Etop-MNPs retained their cytotoxic effect after being subjected to the magnetic forces causing MNP rotation and translational movement. The 15 cm pull position was used for the tray position with respect to the magnet, as in the MNP velocity tests described above. Cellular viability was determined using trypan blue exclusion.

Creation of Etop-MNPs with increasing quantities of biotinylated etoposide was found to correlate with increasing cytotoxicity after MNP translation, in a dose-dependent fashion. Results are shown in Figure 7C, for 1X, 2X, and 5X particles compared to untreated, DMSO, and base MNP controls (D283 cells, 24 h treatment time). Etop-MNPs with the standard (1X) formulation killed 28.7 ± 1.3% of D283 cells; the 5X formulation doubled this, showing a 50% greater cytotoxic effect, after remote delivery (i.e., translational movement down the lanes of the acrylic tray). Similar results were seen with H2122 cells analyzed by MTT assay and trypan blue exclusion, after only 2 h of treatment. These results are shown in Figure 7D. Viability of H2122 cells was found to be 66.6 ± 2.3% after only 2 h of treatment with Etop-MNPs (by trypan blue exclusion). Base MNPs did not have this toxic effect on the cells. Therefore, Etop-MNPs demonstrated a killing effect on cancer cells which was dose-dependent. This chemotherapeutic effect was retained after translational movement on a human-sized scale, as generated by a remote (rotating) permanent magnet.

## Discussion

These findings indicate that etoposide-laden MNPs can be easily produced, moved remotely using a rotating magnet over human-sized distances, and produce a cytotoxic effect even after translational movement. The advantages of using Etop-MNPs with magnetic propulsion over etoposide alone are that it can be moved to target areas much more rapidly, even against bulk CSF flow, and that the drug is far less diluted (16). Etoposide can be released along the delivery path of the Etop-MNPs, as they preferentially cling to cancer cells (16). Etop-MNPs utilize the streptavidin (SA) -biotin interaction, which has long been used to great advantage in a variety of biological applications. Here, D283 and H2122 cells were used to represent medulloblastoma and non-small cell lung cancer, respectively, two types of cancer notorious for dissemination through CSF pathways. Glioblastoma cells (U138) were also tested to confirm the results; glioblastoma can spread through CSF as well. Leptomeningeal spread is problematic in patients with many types of cancer, especially children with Group 3 and 4 medulloblastoma (29–33).

Etoposide forms a ternary complex with DNA and topoisomerase II, inducing breaks in double -stranded DNA which prevent entry into mitosis and lead to cell death (34–36). While etoposide and related drugs are important, effective, and widely-used cancer agents, they have significant toxic side effects when administered systemically (23, 36). CSF penetration of systemically-administered etoposide is poor (9, 35). Intrathecal/intraventricular administration of etoposide dramatically increases CSF drug levels in comparison with IV administration, thus improving efficacy. Etoposide has previously been incorporated into non-magnetic nanoparticles and MNPs by a different method, with testing on cell lines including U87 (37–41).

Intrathecally-administered agents (whether given by the lumbar or ventricular route) are only expected to permeate tissue to a depth of a few millimeters, and therefore could only rationally be used in LM patients with non-bulky disease (1, 42, 43). Use of Etop-MNPs would improve upon this situation because they could be used to focally increase drug delivery, even to areas that represent “blind alleys” with respect to CSF access (16). Iron oxide particles have been administered intrathecally in animals (44–47). Nanostructures have been proposed for enhancing intrathecal drug delivery (16, 43–45). A variety of chemotherapeutic agents can be bound to, and transported by MNPs (12, 16) MNP-chemotherapy constructs have shown growth-inhibitory effects *in vitro* and in animal studies for several types of cancer cells, including glioblastoma (12, 16). Tumor cells are likely to demonstrate heightened non-specific binding and uptake of drugs and nanoparticles due to their greater metabolic needs (12, 16).

The advantage of MDT lies in its potential ability to control the delivery and concentration of a drug at desired locations, while minimizing toxicity in untargeted tissue (16, 18, 19). Movement of MNPs within human CSF pathways is significantly more problematic than in animals due to the distances involved (16, 18). A solution may be to use magnet rotation or an oscillating field for propulsion. Potential clinical applications for rotational MDT have recently been highlighted, and this principle may offer advantages for other fluid filled spaces within the body as well (16, 18, 19). Animal studies have been reported which utilize the rotational mechanism (16, 19). While Etop-MNPs may not penetrate solid tissue, they should be able to be directed through conduits such as a patent spinal subarachnoid space, primarily limited by surface adherence (16, 48).

Certainly our results are from a simple model system; the situation with CSF in humans is far more complex. Our intent in this paper is to provide the first description of these Etop-MNPs, which were created using a novel method. Data presented here pertain to Etop-MNP characterization and *in vitro* testing; the next phase is to explore the movement and imaging of Etop-MNPs in an animal model. While further studies are required to safely bridge the gap between preclinical experiments and patient use, an improved ability to direct chemotherapy (and other drugs) within CSF pathways would represent a major step forward in the treatment of patients with LM and other diseases.

## Data Availability Statement

The raw data supporting the conclusions of this article will be made available by the authors, without undue reservation.

## Author Contributions

All authors have contributed to the experimental design, implementation, analysis and interpretation of the data, and/or been involved in the writing of the manuscript.

## Conflict of Interest

SH and BL were employees of IMRA America, Inc. during the time this research was conducted. SM is an employee of Pulse Therapeutics, Inc. The remaining authors declare that the research was conducted in the absence of any commercial or financial relationships that could be construed as a potential conflict of interest.
